# Thermal Processing and Fortification with Ayocote and Quintonil Flours Affect Blue Maize Tortilla and Tortilla Chip Properties

**DOI:** 10.3390/molecules31162774

**Published:** 2026-08-10

**Authors:** Edwin Rojo-Gutiérrez, Leticia Xochitl López-Martínez, Mónica Alejandra Villegas-Ochoa, Ezequiel Muñoz-Márquez, Ramiro Baeza-Jiménez

**Affiliations:** 1 Laboratorio de Biotecnología y Bioingeniería, Centro de Investigación en Alimentación y Desarrollo (CIAD), A.C., Av. Cuarta Sur 3820, Fracc. Vencedores del Desierto, Delicias CP. 33089, Chihuahua, Mexico; edwin.rojo@ciad.mx; 2 Laboratorio de Antioxidantes y Alimentos Funcionales, SECIHTI, Centro de Investigación en Alimentación y Desarrollo (CIAD), A.C., Carretera Gustavo Enrique Astiazarán Rosas No. 46, Col. La Victoria, Hermosillo CP. 83304, Sonora, Mexico; 3 Coordinación de Tecnología de Alimentos de Origen Vegetal, Centro de Investigación en Alimentación y Desarrollo (CIAD), A.C., Carretera Gustavo Enrique Astiazarán Rosas No. 46, Col. La Victoria, Hermosillo CP. 83304, Sonora, Mexico; mvillegas@ciad.mx; 4 Laboratorio de Fisiología y Nutrición Vegetal, Centro de Investigación en Alimentación y Desarrollo (CIAD), A.C., Av. Cuarta Sur 3820, Fracc. Vencedores del Desierto, Delicias CP. 33089, Chihuahua, Mexico; emunoz@ciad.mx

**Keywords:** *Amaranthus hybridus*, *Phaseolus coccineus*, bioaccessibility, underutilized vegetable species, antioxidant activity, texture, staple food

## Abstract

Modern food systems face the challenge of improving nutritional quality while reducing environmental impact and conserving agrobiodiversity. This study evaluated the effects of thermal processing and fortification with ayocote (*Phaseolus coccineus*) and quintonil (*Amaranthus hybridus*) flours on the physicochemical, nutritional, and functional properties of blue maize tortillas, baked tortilla chips (BTCs), and fried tortilla chips (FTCs). Two nominal fortification levels (F1: 6% and F2: 9%) were incorporated into nixtamalized blue maize flour. The resulting products were characterized for proximate composition, mineral profile, color, texture, phenolic and flavonoid contents, antioxidant activity, and in vitro phenolic bioaccessibility. Fortification significantly increased protein, ash, mineral, phenolic, and flavonoid contents, particularly in F2 formulations, while maintaining adequate technological quality. Thermal processing influenced phytochemical content and antioxidant activity, although these effects depended on the parameter evaluated. BTCs generally exhibited higher flavonoid content and antioxidant capacity, whereas total phenolic content remained comparable between BTCs and FTCs. Fortification improved tortilla flexibility but increased tortilla chip hardness. During simulated gastrointestinal digestion, fortified products released more phenolics than the controls. BTC-F2 maintained the highest phenolic content after digestion, whereas tortilla formulations exhibited the greatest relative phenolic bioaccessibility. These findings demonstrate the potential of the underutilized native species ayocote and quintonil to enhance the nutritional and functional value of maize-based products.

## 1. Introduction

Current challenges in food systems include the need to improve nutritional quality, reduce environmental impact, and preserve agrobiodiversity [1,2]. In this regard, underutilized plant species have emerged as promising candidates due to their high nutritional value, resilience to climatic stress, and potential to diversify agricultural systems and diets [3,4]. Recent studies have highlighted that these species are rich sources of proteins, dietary fiber, essential minerals, and bioactive compounds, including phenolic acids, flavonoids, and other antioxidants, which have been associated with reduced risk of chronic diseases and improved metabolic health [1,3,5]. Despite these advantages, their integration into modern food systems remains limited, primarily due to the dominance of monoculture systems that prioritize high-yielding staple crops over diverse native species. This situation is further intensified by economic and policy constraints, including underdeveloped markets, weak value chains, and limited institutional support. In addition, sociocultural perceptions and technological limitations continue to restrict their adoption, use, and broader dietary incorporation [6].

Among these resources, blue maize (*Zea mays* L.), ayocote (*Phaseolus coccineus* L.), and quintonil (*Amaranthus hybridus* L.) represent native Mexican species with significant nutritional and functional potential. Blue maize has been widely reported to contain elevated concentrations of anthocyanins and hydroxycinnamic acids, compounds associated with strong antioxidant and anti-inflammatory activities [7]. Similarly, ayocote beans are recognized for their high protein content and diverse phenolic profile, including flavonoids and phenolic acids that contribute to their functional properties [8,9]. Quintonil, a traditional quelite, has also been characterized as a valuable source of micronutrients and bioactive compounds, with reported antioxidant, antihypertensive, and hypoglycemic activities [10,11]. Nonetheless, these species remain underexploited in contemporary diets, thereby limiting their potential to improve dietary quality and sustainability.

The fortification of staple foods has been widely recognized as an effective and scalable strategy to improve population-level nutrient intake, particularly in regions with limited dietary diversity [12]. In Mexico and other Latin American countries, maize-based foods such as tortillas and tortilla chips constitute a fundamental component of daily diets, providing substantial caloric and protein intake [13,14]. Their widespread consumption, cultural acceptance, and technological adaptability make them ideal matrices for incorporating functional ingredients. In addition, pigmented maize varieties, such as blue maize, offer intrinsic functional advantages due to their phytochemical composition, which may complement the properties of added plant ingredients to enhance the nutritional and antioxidant profile of derived products. While tortillas have been extensively studied as vehicles for fortification [15,16,17,18], processed derivatives such as tortilla chips remain comparatively underexplored, particularly with respect to incorporating combined plant matrices, including legumes and leafy vegetables.

However, incorporating plant-based ingredients into maize systems introduces complex physicochemical interactions that may affect both product quality and functional properties [19,20]. Thermal processing, inherent to the preparation of tortillas and especially chips (which often involve baking or frying), plays a critical role in determining the stability and bioaccessibility of bioactive compounds. It is well known that heat treatments can induce structural modifications in starch–protein–fiber networks, influence the release of bound phenolic compounds, and alter antioxidant activity. In some cases, processing may reduce measurable phenolic content due to degradation or interactions with dietary components, such as fiber or proteins, which limit extractability [21]. Conversely, gastrointestinal digestion (particularly under acidic gastric conditions) can promote the release of bound phenolics, increasing their bioaccessibility, whereas neutral intestinal conditions may lead to their transformation or reduced stability [22,23]. These contrasting effects highlight the importance of evaluating both processing and digestion to accurately determine the functional potential of fortified foods.

Although previous studies have evaluated fortifying maize products with individual plant ingredients, there remains limited understanding of interactions among combined matrices derived from underutilized species, particularly in pigmented maize products. Moreover, few studies have simultaneously examined how formulation and thermal processing jointly affect both the nutritional composition and the bioaccessibility of phenolic compounds using in vitro digestion models. To the best of our knowledge, no previous study has comprehensively addressed these factors in combination within blue maize-based systems incorporating ayocote (AF) and quintonil (QF) flours. Understanding these limitations could help clarify the possible interactions among the incorporated ingredients and support the future development of functional staple foods.

Based on the nutritional composition and bioactive profiles of both plant species, we hypothesized that: (i) fortification with ayocote and quintonil flours would improve the nutritional composition and enhance the phenolic content and antioxidant activity of blue maize tortillas and tortilla chips; and (ii) thermal processing would differentially affect phytochemical retention and bioaccessibility, with baking preserving these compounds more effectively than tortilla cooking or frying.

Therefore, the aim of this study was to obtain blue maize tortillas and tortilla chips fortified with AF and QF, and to systematically evaluate the effects of formulation and thermal processing on their nutritional properties, textural properties, phenolic retention, antioxidant capacity, and bioaccessibility. This approach aims to contribute to the revalorization of underutilized native species and to support the development of nutritionally enhanced staple foods within sustainable, culturally relevant food systems.

## 2. Results and Discussion

### 2.1. Nutritional Enhancement Through Fortification

Moisture content showed clear differences (*p* ≤ 0.05) among treatments, as expected from the applied thermal processes. Tortillas exhibited the highest moisture values (~50–51%), followed by FTCs (~6.18–9.55%) and BTCs (~6.59–6.75%). This tendency was consistent, given the dehydration during baking and frying. No significant differences were observed among tortilla and BTC formulations, indicating that fortification at 6–9% did not substantially affect water retention in the nixtamalized matrix. In contrast, FTC samples showed a progressive increase in moisture with fortification. This may be associated with the higher water-binding capacity of fiber- and protein-rich ingredients such as ayocote and quintonil [24,25,26], as well as with frying dynamics. It is known that rapid surface crust formation may limit water diffusion in fried foods, promoting moisture retention within the product [27]. Such effects are less pronounced in tortillas, due to their high initial moisture content, and in BTCs, where dehydration occurs more gradually without the formation of a frying-induced crust.

Protein content increased significantly (*p* ≤ 0.05) with fortification across all treatments, confirming the nutritional contribution of AF and QF. In tortillas, protein content increased from 7.98% in the control to 10.43% in F2, representing approximately a 31% increase. A similar trend was observed in BTCs, where fortified samples reached values above 10%. In FTCs, protein content also increased with fortification; however, values remained significantly lower than those observed in BTCs but were partially comparable to those of tortillas. This could be related to the dilution effect of oil uptake during frying. These fortification results are consistent with previous studies demonstrating that incorporating legume flours enhances protein content in cereal-based products [28,29]. Similar increases have been reported in previous work on conventional maize tortillas fortified with ayocote–quintonil blends (9%, 2:1 ratio), with protein content increasing by up to 29.4% [26]. Likewise, Sánchez-Villa et al. [30] reported approximately a 30% increase in protein content in tortillas fortified with *Phaseolus coccineus* protein concentrate (10%). Higher protein levels (14.78 g/100 g) have also been reported in BTCs formulated with *Phaseolus vulgaris* L., where a substantially higher partial substitution of maize flour (up to 45%) was employed [28], which may explain the greater protein enrichment observed. Astorga-Gaxiola et al. [31] developed blue maize tortillas fortified with quelite (*A. hybridus*) at substitution levels up to 30%, resulting in a protein content of 10.33%, which is comparable to the highest values obtained in the present study, despite lower substitution levels being used (6–9%). Lipid content was significantly influenced by the treatment’s thermal processing. As expected, FTC formulations exhibited significantly higher lipid levels (13.90–16.24%) than BTCs (2.70–3.61%) and tortillas (~1.39–1.76%), reflecting oil uptake during frying. As shown in Table 1, the lipid values of BTC formulations were significantly higher than those observed in tortillas. It is possible that the higher lipid values observed in BTCs resulted from structural modifications induced by baking, which may have increased lipid extractability by disrupting starch–protein–lipid interactions and increasing matrix porosity, thereby enhancing solvent access during extraction [32]. In BTCs, fortified formulations exhibited lower lipid values than the control, which may be attributed to a dilution effect due to the partial replacement of maize flour with ayocote-quelite blends. Similar effects have been reported in other studies of tortillas and tortilla chips fortified with low-fat ingredients, such as broccoli, beans, quelite, and apple peel [15,26,30,31,33,34]. A similar tendency was observed in tortillas and FTC-fortified formulations; however, only the tortilla-F1 and FTC-F2 samples showed a statistically significant reduction in lipid content compared to their controls (*p* ≤ 0.05). Overall, lipid content was reduced by up to 21%, 22%, and 14% in tortillas-F2, BTC-F2, and FTC-F2, respectively, compared to their control formulations. It is worth noting that the lipid content of tortillas in the present study was lower than that reported by Sánchez-Villa et al. [30] (2.50–2.81%) and Astorga-Gaxiola et al. [31] (2.17–2.71%), who also fortified tortillas with ayocote (protein concentrate) and quelite. In contrast, the lipid content of BTCs was consistent with values reported by Rodríguez-Miranda et al. [28], who developed fortified BTCs using red bean (*Phaseolus vulgaris* L.) and beet (*Beta vulgaris*) flours, with lipid contents ranging from 2.91 to 3.80%. Similarly, FTC lipid values were comparable to those reported by Amador-Rodríguez et al. [35], who formulated fortified FTCs using soy and *Opuntia* flours at fortification levels between 4% and 10%, obtaining lipid contents in the range of 12.96–16.67%.

Ash content increased significantly with fortification across all treatments, indicating an enhanced mineral contribution from AF and QF. Since ash content is considered an indicator of mineral density, these results highlight the potential of both ingredients as natural alternatives for improving the micronutrient profile of staple foods. Relative to the control, ash values increased by approximately 69% in FTCs, 49% in tortillas, and 45% in BTCs. These results are consistent with previous reports highlighting the high mineral content of underutilized plant species and their potential to improve micronutrient density in staple foods [8,36,37,38]. Although fortified FTC samples exhibited the largest relative increases compared with their controls, tortillas and BTC treatments achieved significantly higher ash values. Carbohydrate content showed an inverse relationship with protein and ash, decreasing as fortification levels increased. This dilution effect is expected because the starch-rich blue maize flour is partially replaced by higher-protein, mineral- and fiber-rich ingredients [31,39,40], thereby lowering the carbohydrate contribution. Crude fiber content was significantly affected by both formulation and processing (*p* ≤ 0.05). In tortillas, fiber values decreased with fortification, from 2.50% (C) to 2.11% (F1) and 2.21% (F2). A similar trend was observed in FTCs, where the F2 formulation presented lower fiber content than the control. In contrast, BTC samples showed an increase in crude fiber with increasing fortification, with the F2 formulation reaching the highest value (3.18%). These results indicate that the effect of fortification on crude fiber is highly dependent on the processing method. The decrease observed in tortillas and FTCs may be attributable to limitations of the crude fiber method, which does not account for all dietary fiber fractions and may be influenced by matrix interactions or structural modifications induced by hydrothermal processing [41]. Conversely, the increase in BTCs is likely linked to structural changes during baking, such as dehydration and matrix rearrangement, which enhance the retention or measurement of insoluble fiber components [28].

Caloric values reflected the combined effects of proximal composition and processing. Tortillas exhibited the lowest caloric content (~200 kcal/100 g), whereas BTCs (~376 kcal/100 g) and, especially, FTCs (~421.4 kcal/100 g) showed significantly higher values due to moisture reduction and lipid incorporation. No significant differences were observed among formulations within the same treatment. Nevertheless, fortified formulations generally exhibited lower lipid and carbohydrate contents and higher protein and ash levels, suggesting an improved nutritional profile compared to their respective controls.

Overall, the results demonstrate that incorporating AF and QF enhances the nutritional composition of blue maize-based products by increasing protein, mineral, and fiber contents, while modulating lipid contribution and caloric intake, depending on the processing method. These findings reinforce the potential of underutilized native species as functional ingredients to improve the nutritional quality of staple foods, consistent with recent literature emphasizing the role of plant-based fortification strategies in sustainable food systems [42].

Mineral quantification revealed that both formulation and thermal processing significantly influenced the mineral profile of the obtained products. As for the proximate composition results, fortified tortillas and chips generally exhibited higher mineral contents (Table 2), where formulation F2 can be distinguished in all the treatments. BTCs exhibited the greatest mineral profile modifications, followed by FTCs and tortillas, highlighting the effect of the thermal processes. With respect to their corresponding controls, calcium (Ca), potassium (K), magnesium (Mg), and manganese (Mn) contents increased by approximately 12%, 65%, 55%, and 65%, respectively. In contrast, the contents of iron (Fe), sodium (Na), and zinc (Zn) decreased by an average of 29%, 65%, and 20%, respectively. These findings suggest that AF and QF can substantially modify the mineral composition of nixtamalized maize products depending on the formulation and processing conditions applied. It is important to note that nixtamalized maize products are already recognized as dietary sources of minerals, particularly Ca, due to the incorporation of Ca(OH)_2_ during nixtamalization. Although some minerals, such as Fe and Zn, decreased, incorporating the vegetable species produced a more balanced and functional product and an enhanced mineral profile, accompanied by a substantial reduction in Na content. Similar mineral enrichment effects have been observed in maize-based products fortified with alternative vegetable-derived flours [28,31].

### 2.2. Color Characteristics

The colorimetric parameters revealed that both formulation and thermal processing significantly influenced the optical properties of the blue maize-based products (Table 3), suggesting complex interactions among native pigments, added plant matrices, and heat-induced reactions. Tortillas exhibited the highest luminosity (L*) values (~40–44), whereas FTCs showed the lowest (~28). The marked darkening observed in FTCs might be associated with Maillard reactions, lipid absorption, and thermal degradation/polymerization of phenolic compounds during frying [43,44]. Fortified formulations reduced a* values in tortillas and FTCs, indicating lower redness, likely due to dilution/degradation of anthocyanins and incorporation of dark green pigments from the quelite. In contrast, BTCs exhibited higher values after the fortification. This contrast may indicate that tortilla chips undergo greater thermal degradation or structural modification of anthocyanin-related pigments during frying than during baking. This interpretation is supported by the fact that, despite chroma (C*) values increasing substantially after fortification across all treatments, color intensity followed the order: BTC > tortilla > FTC. These findings suggest that dry-heat processing better preserves thermolabile compounds than frying. The hue angle (h°) decreased significantly as thermal intensity increased, resulting in a color shift from yellowish toward darker reddish-brown tonalities. with tortillas exhibiting the highest values (~85–86), whereas FTCs had the lowest (~45–60). Conversely, the partial substitution of blue maize flour with AF and QF shifted the chromatic profile toward yellow-green regions. This effect was also observed in a previous research in which fortified tortillas were enriched with AF and QF [26].

### 2.3. Textural Properties of Masa, Blue Maize Tortilla, and Tortilla Chips

The incorporation of quintonil and ayocote beans significantly reduced the hardness of the maize masa (Table 4). Both fortified formulations exhibited lower hardness than the control (no significant difference between them), indicating a softer, more deformable masa matrix. Adhesiveness was also significantly influenced by formulation. F1 exhibited the highest negative adhesiveness value (−1.16), followed by F2 (−0.8), whereas the control showed the lowest adhesiveness (−0.23). Adhesiveness describes the work required to overcome the attractive forces between the food surface and another material and is therefore closely associated with masa stickiness and handling properties. Despite the increase in adhesiveness, masas maintained adequate machinability during manual pressing and handling. In contrast, cohesiveness and elasticity did not differ significantly among formulations, suggesting that fortification did not adversely affect the masa’s internal structural integrity or viscoelastic recovery properties. These results indicate that incorporating AF and QF produced softer and more adhesive masas while preserving adequate structural functionality for tortilla and tortilla chips processing. A similar behavior was observed in our previous study [26], supporting the reproducibility of these textural properties.

Tortilla’s mechanical behavior was also significantly influenced by the incorporation of AF and QF (Table 5). F2 exhibited the highest tensile strength (2.22 N), whereas F1 showed values comparable to the control formula. F2 tensile strength is very similar to that obtained when the same formulation was utilized to supplement white maize tortillas [26]. This result supports the previous hypothesis that legume-derived proteins and polysaccharides act as structuring agents, reinforcing the tortilla matrix and improving its mechanical resistance. On the other hand, extensibility increased (*p* ≤ 0.05) in both fortified formulations, with F1 exhibiting the highest value (15.97). The addition of blended plant matrix flours may increase fiber content. Increased fiber levels are commonly associated with softer tortilla textures, likely due to a reduction in the proportion of gelatinized starch within the matrix. In addition, the presence of fiber particles may interfere with starch retrogradation, limiting the development of a continuous crystalline network and consequently contributing to a more flexible tortilla structure [45]. Regarding rollability, no fracture was observed during rolling (1), demonstrating that fortification did not affect tortilla flexibility or handling properties. Rollability is considered a critical quality attribute because it reflects tortillas’ ability to maintain structural integrity during folding and rolling operations, a characteristic closely associated with consumer acceptability and technological functionality. These results are consistent with our previous findings in conventional maize tortillas fortified with AF and QF [26].

The texture of tortilla chips was significantly affected (*p* ≤ 0.05) by both formulation and processing method (Table 6), confirming the influence of thermal treatment on the products’ mechanical behavior. BTCs generally exhibited higher fracturability than FTCs, whereas higher hardness values were observed only for the control and F1 formulations. Fortification with AF and QF increased chip hardness under both processing methods; however, in BTC, only the F1 formulation exhibited significantly higher hardness than the control, reaching the maximum value of 2436.38 g. This behavior suggests that moderate levels of AF and QF incorporation favored the development of a firmer product structure, possibly by promoting stronger interactions among starch, proteins, fiber, and non-starch polysaccharides, thereby contributing to the greater hardness observed in F1 [46]. In contrast, although F2 contained higher AF levels, excessive incorporation may have altered the structural organization of the product due to the presence of polysaccharides and other non-cellulosic compounds, thereby influencing its textural properties [47]. This behavior contrasts with that observed in tortillas, where F2 exhibited greater tensile strength, suggesting that the effect of fortification depends on the structural state generated by processing. While tortillas remain hydrated and flexible systems, tortilla chips are dehydrated and brittle products, in which higher levels of non-starch components may influence texture differently. Regarding fracturability, BTC formulations exhibited the highest values (*p* < 0.05), whereas fortified formulations of each treatment showed decreased fracture distances, indicating that less deformation was required before rupture and suggesting the formation of a more rigid and brittle structure after fortification. This effect may be associated with the disruption of the continuous starch matrix by protein and fiber-rich particles during dehydration. FTCs exhibited lower overall fracturability values than BTCs, and there were no significant differences among the supplemented formulations, suggesting that frying reduced the influence of formulation on fracture behavior, possibly due to partial matrix plasticization from oil uptake. Similar texture effects were observed by Amador-Rodríguez et al. [35], who used *Opuntia ficus-indica* L. and defatted soybean flours to fortify tortilla chips. As they increased the amount of *Opuntia* flour for tortilla production, less force was required to fracture the tortilla chips. Conversely, the defatted soybean flour exerted the opposite effect. However, the combination of both *Opuntia* and soybean flours (~1:1) decreased the fracture resistance of the tortilla chips. Their findings also indicated that increasing oil content reduced chip hardness. These results highlight the important role of processing conditions in determining the mechanical characteristics of fortified tortilla chips.

### 2.4. Phenolic Content Retention, Antioxidant Activity, and In Vitro Bioaccessibility

The phenolic content and antioxidant activity of the processed products depended strongly (*p* ≤ 0.05) on both formulation and processing conditions (Table 7). In general, fortification with AF and QF increased total phenolic content (TPC), total flavonoid content (TFC), and antioxidant activity for all of the treatments. These results align with previous studies, showing that fortifying tortillas and tortilla chips with plant-based ingredients, such as legumes and leafy vegetables, enhances TPC and antioxidant activity by incorporating additional bioactive compounds [26,31,48,49]. Among the processed products, tortillas generally exhibited lower TPC values than BTCs and FTCs, with the exception of the control formulation, particularly in formulation F1, suggesting that tortilla cooking may have promoted greater degradation and leaching of soluble phenolic compounds than baking or frying [49]. However, the TPC (73 mg GAE/100 g ds) and TFC (93 mg QE/100 g ds) exhibited by F2 in the present research are comparable to or higher than those obtained by Asgorga-Gaxiola et al. [31], who reported a TPC and TFC of 73 mg GAE/100 g and 70 mg QE/100 g, respectively, from their tortillas with 20% of quelite. TPC generally increased with fortification level, with F2 exhibiting the highest values in masa (105 mg GAE/100 g ds), BTCs (100 mg GAE/100 g ds), and FTCs (102 mg GAE/100 g ds). These results indicate that TPC values in BTCs and FTCs remained comparable to those of fortified masa, particularly in formulation F2. Interestingly, any significant differences (*p* ≥ 0.05) in TPC were observed between BTCs and FTCs within the same formulation, suggesting that both thermal treatments resulted in comparable levels of extractable phenolic compounds despite their different processing mechanisms. A comparable tendency was observed for TFC. Masa and BTCs exhibited the highest flavonoid concentrations, particularly in fortified formulations (>260 mg QE/100 g ds), whereas tortillas showed the lowest values, especially F1 (39 mg QE/100 g ds). These findings indicate that flavonoids were more susceptible to degradation during tortilla cooking than during baking. The greater TFC values observed in BTCs may be associated with lower moisture conditions and reduced direct water–heat interactions, which likely minimized flavonoid leaching and degradation. FTCs exhibited intermediate TFC values (~132–138 mg QE/100 g ds), suggesting partial flavonoid losses caused by oxidation and thermal instability of anthocyanin-related compounds during frying. Unlike TPC, TFC differed significantly among treatments, with BTCs being the only processed product that exhibited flavonoid levels comparable to those of masa. Thus, TFC preservation followed the order: baking > frying > cooking. The TPC and TFC values obtained for BTCs in the present study were higher than those previously reported for tortillas fortified with quelite (83 mg GAE/100 g and 123 mg QE/100 g, respectively, [31]) and quelite-ayocote blends (93 mg GAE/100 g ds, [26]). However, lower values were observed compared to those reported by Vasisht et al. [49], who developed FTCs formulated with germinated corn, mung bean flour, and pomegranate peel, reaching 30,785 mg GAE/100 g and 59 mg QE/100 g, respectively.

Antioxidant activity, as determined by DPPH and ABTS, generally followed the same pattern as TPC and TFC (Table 7). Fortified masa and BTCs exhibited the highest antioxidant activities, with DPPH values above 2.10 mg TE/g ds and ABTS values reaching up to 3.25 mg TE/g ds in BTC-F2. These findings suggest that baking preserved antioxidant compounds more effectively than tortilla cooking or frying. In tortillas, antioxidant activity decreased considerably after processing, particularly for DPPH, which may indicate degradation of thermolabile antioxidants during cooking. FTCs exhibited the lowest ABTS values (~1.51–1.62 mg TE/g ds), suggesting that frying had a stronger negative effect on radical-scavenging compounds due to oxidation promoted by high temperatures and oil exposure. Similar behaviors have been reported in fortified maize matrices, where thermal processing altered phenolic compound stability and antioxidant performance depending on interactions among TPC, proteins, and dietary fiber [49]. Overall, TPC remained comparable between BTCs and FTCs; however, BTCs exhibited higher flavonoid content and antioxidant activity, suggesting that baking not only better preserves thermolabile phytochemicals than tortilla cooking or frying, but also maintains phytochemical levels comparable to those of fortified masa. These results demonstrate that incorporating AF and QF increased bioactive compound content while preserving their functional antioxidant capacity, consistent with previous studies on fortified tortillas and tortilla chips (Table 8) [31,46,49,50].

Phenolic compounds release during simulated gastrointestinal digestion followed different trends depending on the applied thermal treatment and fortification level. In general, fortified formulations exhibited higher phenolic release (*p* ≤ 0.05) than their respective controls, demonstrating that AF and QF contributed additional bioactive compounds to the maize matrix. For all treatments, TPC increased from the undigested to the oral phase, then increased more pronouncedly during gastric digestion, followed by a subsequent reduction under intestinal conditions. The initial increase observed during the oral phase may be associated with sample matrix hydration and the action of salivary enzymes, which could facilitate the release of readily extractable or weakly bound phenolic compounds from the food matrix [51]. TPC increased (*p* ≤ 0.05) in all of the treatments from the oral to the gastric phase, exhibiting the highest phenolic content release, particularly in BTC-F2 (273 mg GAE/100 g ds) and tortilla-F2 (244.42 mg GAE/100 g ds), likely due to acidic hydrolysis and enzymatic disruption of the matrix, which further enhanced the release of bound phenolic compounds remaining in the extracts [26]. After intestinal digestion, TPC decreased considerably (*p* ≤ 0.05) in all of the treatments, likely due to phenolic compound instability under intestinal conditions and interactions with proteins or dietary fiber [52]. Nevertheless, fortified BTC and FTC formulations, especially F2, maintained the highest intestinal TPC values (~61–63 mg GAE/100 g ds), suggesting greater preservation of bioaccessible phenolic compounds after baking and frying. Although differences between BTCs and FTCs varied throughout digestion, both processing methods exhibited comparable TPC values despite their distinct processing conditions, most notably in the F2 formulation. Similar behavior was previously observed in our work on conventional maize tortillas fortified with ayocote and quintonil flours [26]. However, although gastric digestion promoted the greatest release of phenolic compounds in both studies, our previous work on conventional fortified tortillas showed a different release pattern (gastric > intestinal > oral). The different phenolic content release pattern observed in the present study may be associated with differences in the phenolic composition of blue maize, particularly its anthocyanin profile. Previous studies on blue maize have demonstrated that anthocyanins undergo characteristic transformations during simulated gastrointestinal digestion, resulting in release and stability patterns that differ from those reported for the phenolic compounds predominant in non-pigmented maize [53]. These differences may partially explain the distinct phenolic release pattern in the present study. Additionally, the intestinal TPC values previously reported for F1 formulation (22 mg GAE/100 g ds) and F2 formulation (73 mg GAE/100 g ds) were comparable to those obtained in the present work.

While the TPC values discussed above reflect the analytical release of extractable phenolic compounds during simulated gastrointestinal digestion, the bioaccessibility index (BI) provides complementary information by estimating the proportion of phenolic compounds that remain bioaccessible following the intestinal phase.

The BI also differed among treatments. Tortilla-F1 exhibited the highest BI value (93.54%), followed by tortilla-F2 (71.63%) and BTC-F2 (65.03%). However, it is important to consider that BI represents a relative percentage rather than the absolute amount of bioaccessible phenolic compounds. Thus, formulations exhibiting higher initial TPC values may still provide greater phenolic content availability after digestion despite presenting comparatively lower BI values. This behavior was particularly evident in BTC-F2, which maintained the highest phenolic content throughout digestion, even though its BI was lower than that of the tortilla formulations. Compared to previous studies, the BI values observed here (38.24–93.54%) are lower than those reported for several blue maize tortillas, where total phenolic bioaccessibility frequently exceeded 100% and in some cases reached values above 390% and even 900% [54,55], likely due to the intestinal release of matrix-bound phenolics during digestion [56]. However, these values are still consistent with the variability (30–86.84%) reported for cereal products [26,57], since bioaccessibility is strongly influenced by processing conditions, food matrix, and the chemical stability of individual compounds [58]. Therefore, a lower BI should not be interpreted as a lower delivery of phenolics *per se*, because formulations with higher initial phenolic contents may still provide greater absolute amounts of bioaccessible compounds after digestion [57,59]. The higher BI values observed in tortillas may also be associated with their softer, more hydrated structure, which could facilitate enzymatic accessibility and the release of phenolic compounds during digestion [60].

## 3. Materials and Methods

### 3.1. Plant Materials and Chemical Reagents

Nixtamalized blue maize flour (Naturelo^®^) was purchased from Amazon México. Ayocote beans (*Phaseolus coccineus*) were sourced from a local market in Toluca, Estado de México, México, whereas quintonil (*Amaranthus hybridus*) was collected in Delicias, Chihuahua, México. Both raw materials were washed with potable water, then dried at 40 °C for 12 h in an incubator (Felisa model FE-133D; Felisa^®^, Zapopan, Jalisco, México). The dried samples were subsequently milled using a conventional grinder (IKA A11 basic mill, IKAWorks, Inc., Wilmington, NC, USA) and passed through a 500 µm sieve. The flour passing through the sieve was collected, stored in airtight polypropylene bags (Ziploc^®^, S. C. Johnson & Son, Inc., Racine, WI, USA), and kept in a desiccator at room temperature until further use. All reagents and solvents used were analytical grade. Standards for phenolic and flavonoid compound determination, as well as antioxidant and in vitro digestion assays, were purchased from Sigma-Aldrich (St. Louis, MO, USA).

### 3.2. Blue Maize Tortillas and Tortilla Chips Production

Blue maize tortillas and tortilla chips (baked and fried) were formulated using two nominal fortification levels (6% and 9%), obtained by incorporating ayocote-quelite flour blends into nixtamalized blue maize flour (Naturelo^®^), as shown in Table 9. F1 consisted of a nominal 6% fortification (actual inclusion: 6.25% *w*/*w*) using a 1:1 ayocote:quintonil blend, whereas F2 consisted of a nominal 9% fortification (actual inclusion: 8.75% *w*/*w*) using a 2:1 ayocote:quintonil blend. Throughout the manuscript, F1 (6%) and F2 (9%) refer to these nominal target fortification levels.

Tortillas were prepared following a previously reported procedure [20]. Briefly, approximately 30 mL of distilled water was added to each flour formulation to obtain a homogeneous masa, which was allowed to rest in a sealed container for 30 min. Approximately 39.8 g of masa was then pressed into tortillas (14 cm in diameter and 1.9 mm thick) using a commercial tortilla press (KUYYFDS, manufactured in China and acquired from Amazon MEX, Mexico City, Mexico). The tortillas were cooked directly on a preheated cast-metal griddle (Tramontina^®^, Carlos Barbosa, Brazil) maintained at a surface temperature of 280–300 °C, as monitored using a digital infrared thermometer (RZ lightning Co., Shenzhen, China). Each tortilla was cooked for 30 s on the first side, 50 s on the second side, and a final 30 s on the first side. For BTCs and FTCs, the masa sheet was prepared using the same procedure as for tortilla production. Each masa sheet (14 cm diameter, ~39.8 g) was cut into 8 triangular portions (~7 cm in length and 5.4 cm at the base) using a 3D-printed custom mold. The 8 tortilla chips obtained from each masa sheet constituted one experimental replicate. For BTC production, the masa triangles were baked in a conventional oven (Proctor Silex^®^, Glen Allen, VT, USA, model 31118PS) at 210 °C for 25 min, with the masa triangles turned every 5 min to ensure uniform heat distribution and even baking. For FTC preparation, the masa triangles were initially baked at 210 °C for 20 min to reduce moisture content (<10%). The pre-baked chips were subsequently tempered at room temperature and then fried in canola oil (Edibel^®^ vegetable oil 1-2-3) at 180 °C for 10 s. After frying, excess surface oil was removed by blotting with paper towels.

All samples were packaged in airtight polypropylene bags (Ziploc^®^) and stored in a desiccator at room temperature until further analysis, including proximate composition, texture, and functional properties.

### 3.3. Chemical Composition Analysis

The proximate composition of maize tortillas, BTCs, and FTCs, including moisture, protein, lipids, ash, and crude fiber, was determined following AOAC methods (925.09, 955.04, 920.39, 923.03, and 985.29, respectively) [61], while carbohydrate content was calculated by the difference according to the following equation:

Carbohydrates (%) = 100% − (moisture + protein + lipids + ash + crude fiber) %(1)

The caloric value (kcal/100 g) was estimated using the Atwater conversion factors (4 kcal g^−1^ for protein, 4 kcal g^−1^ for carbohydrates, and 9 kcal g^−1^ for lipids). Moisture content and caloric values are expressed on a wet basis, whereas all remaining compositional parameters are expressed on a dry matter basis.

### 3.4. Mineral Content

Mineral content was determined according to the method described by Wolf [62]. A 1 g portion of dried sample (blue maize tortilla and tortilla chips) was digested with 25 mL of a triacid mixture composed of HNO_3_ (88.9%), HCl (8.9%), and H_2_SO_4_ (2.2%) in a digestion oven at 300 °C. The digested solution was then diluted to a final volume of 50 mL with distilled water to obtain the stock extract. Concentrations of potassium (K), magnesium (Mg), zinc (Zn), iron (Fe), and manganese (Mn) were quantified using an atomic absorption spectrophotometer (AAS, iCE 3000 Series, Thermo Scientific, Waltham, MA, USA). For the determination of K and Mg, samples were diluted to a 1:100 ratio prior to analysis.

### 3.5. Color Measurement

Color measurements of blue maize tortillas and tortilla chips were performed using a portable colorimeter (Konica Minolta DP-400, Minolta Co., Ltd., Osaka, Japan) following the method described by Páramo-Calderón et al. [63]. Measurements were taken at three locations on the surface of the maize tortilla and chip, with each determination conducted in triplicate. Color parameters were recorded in the CIELAB system (Commission Internationale de l’Éclairage), where L* represents lightness (from black to white), a* indicates the red (+) to green (−) axis, and b* corresponds to the yellow (+) to blue (−) axis. The a* and b* values were then used to calculate chroma (C*) and hue angle (h°).

### 3.6. Capacity of Rollability

Rollability was assessed after tortillas reached room temperature, following the method described by Arámbula-Villa et al. [64]. Each tortilla was wrapped around a stainless-steel rod (2 cm diameter), and the extent of breakage along its length was recorded. Breakage was scored on a scale of 1 to 5, with 1 indicating no breakage (0%), 2 corresponding to 1–25%, 3 to 26–50%, 4 to 51–75%, and 5 to 76–100% of the tortilla length.

### 3.7. Textural Analysis of Masa

Texture profile analysis of masa was conducted using a modified version of the method described by Topete-Betancourt et al. [65]. Masa samples from each formulation were allowed to rest for 30 min, then molded into rectangular prisms (3 × 3 × 2 cm) using a 3D-printed plastic mold. Texture measurements were performed on a TA.XTplus texture analyzer (Stable Micro Systems, Surrey, UK) equipped with a 25 mm diameter cylindrical aluminum probe (P/25). Each sample was placed on the analyzer platform and subjected to a double compression cycle to 40% of its original height at a test speed of 2 mm/s. Texture parameters, including hardness, elasticity, adhesiveness, and cohesiveness, were calculated from the resulting force–time curves.

### 3.8. Textural Analysis of Blue Maize Tortillas

Textural properties of tortillas were evaluated following the method reported by Topete-Betancourt et al. [65], with slight modifications. For each formulation, ten strips (60 × 10 mm) were prepared, and their thickness was measured at ten different points using a micrometer (Mitutoyo, Kobe, Japan). The strips were then subjected to tensile testing using a TA.XTplus texture analyzer (Stable Micro Systems, Surrey, UK) equipped with a 30 kg load cell. Tests were conducted at a crosshead speed of 2 mm/s with an initial grip separation of 4 cm. Tensile strength at fracture (TF, MPa), elongation at break (%E), and Young’s modulus (MPa) were calculated.

### 3.9. Textural Analysis of Tortilla Chips

The fracturability of BTCs and FTCs was measured as proposed by Di Cairano et al. [66] and adapted. A TAXT Plus texture analyzer with a 30 kg cell (Stable Micro Systems, UK) was employed. Hardness was defined as the maximum compression force required to fracture the tortilla chip and was expressed in grams-force (gf). Fracturability was defined as the distance (mm) at which the first major fracture occurred during compression, reflecting the brittleness of the sample. The compression assays were performed using a p/25 cylindrical probe at a descent speed of 1 mm/s and a force of 10 g. For the fracturability analysis, nine replicates were carried out.

### 3.10. Preparation of the Extracts

Extracts were obtained using ultrasound-assisted extraction with a sample-to-solvent ratio of 1:5 (0.25 g/5 mL) and a hydroethanolic solution (25% distilled water and 75% ethanol) as the extraction solvent [67]. Samples were placed in amber vials containing the solvent and sonicated in a water bath (VWR Sonicator model 150 D; VWR International, West Chester, PA, USA) for 30 min at 45 °C. The resulting extracts were filtered through Whatman No. 2 filter paper (Maidstone, UK) and subsequently centrifuged (Eppendorf 5804R, Hamburg, Germany) at 8000 rpm for 15 min. The supernatants were collected and stored in amber vials at 4 °C until further analysis.

### 3.11. Determination of the Total Phenolic Content (TPC)

TPC was determined as described by López-Martínez et al. [67]. Briefly, a 20 µL aliquot of the extract was mixed with 20 µL of Folin–Ciocalteu reagent and allowed to react for 5 min. Subsequently, 20 µL of 0.01 M Na_2_CO_3_ was added, and the mixture was incubated for an additional 5 min. Finally, 125 µL of distilled water was incorporated, and absorbance was measured at 790 nm using a microplate reader (Multiskan Go, Thermo Scientific). Quantification was performed using a gallic acid calibration curve (0–0.2 mg/mL), and results were expressed as mg gallic acid equivalents per 100 of dried sample (mg GAE/100 g ds).

### 3.12. Determination of the Total Flavonoid Content (TFC)

TFC was determined using a colorimetric assay based on the method described by Ghasemi et al. [68]. A total of 200 µL of the extract sample was mixed with 112 µL of deionized water, then 60 µL of methanol and 40 µL of 10% AlCl_3_ were added. Subsequently, 40 µL of 1 M potassium acetate (C_2_H_3_KO_2_) was added, and the mixture was incubated for 30 min at room temperature. Absorbance was measured at 415 nm using a microplate reader (Synergy HT, BioTek Instruments, Winooski, VT, USA). Quantification was performed using a quercetin calibration curve (0–0.2 mg/mL), and results were expressed as mg quercetin equivalents per 100 g of dried sample (mg QE/100 g ds).

### 3.13. Antioxidant Capacity Assays

Antioxidant activity was evaluated using the DPPH and ABTS radical-scavenging assays, following the methods described by López-Martínez et al. [67]. For the DPPH assay, a 60 µL DPPH solution was prepared in methanol. A 7 µL sample of the extract was mixed with 193 µL of DPPH solution, and the mixture was incubated in the dark for 30 min at room temperature. The decrease in absorbance was then measured at 517 nm using a microplate reader (Multiskan Go, Thermo Scientific). Results were quantified using a Trolox calibration curve (0–0.4 mg TE/mL).

For the ABTS assay, the ABTS radical cation (ABTS●^+^) was generated by reacting 7 mM ABTS with 2.45 mM potassium persulfate and allowing the mixture to stand in the dark at room temperature for 16 h. Before analysis, the radical solution was diluted with ethanol to an absorbance of 0.70 ± 0.02 at 734 nm. Subsequently, 10 µL of sample extract were reacted with 190 µL of ABTS radical solution. After 1 min of incubation, absorbance was recorded at 734 nm using the same microplate reader. Antioxidant activity was calculated based on a Trolox standard curve (0–0.7 mg/mL). For both assays, the Trolox-equivalent concentration obtained from the calibration curve was multiplied by the extraction volume (5 mL) and normalized to the dry sample mass. Results were expressed as mg Trolox equivalents per 100 g dry sample (mg TE/100 g ds).

### 3.14. In Vitro Gastrointestinal Digestion of Fortified Blue Maize Tortillas/Tortilla Chips

In vitro gastrointestinal digestion was performed to assess the bioaccessibility of tortillas and tortilla chips. The digestion procedure was based on the standardized static in vitro digestion adapted from Minekus et al. [69]. Tortillas, BTCs and FTCs were subjected to sequential oral, gastric, and intestinal digestion under simulated physiological conditions.

Briefly, for the oral phase, 1 g of tortillas, BTCs and FTCs, (milled as carried out in Section 3.1) was mixed with 3.5 mL of the simulated oral solution (see Table 10), containing α-amylase (75 U/mL) and 25 μL of 0.3 M CaCl_2_; the pH was adjusted to 6.0, and the mixture was incubated at 37 °C for 2 min under agitation (130 rpm). Subsequently, 4 mL of simulated gastric fluid solution (see Table 10), containing porcine pepsin (2000 U/mL) and 5 μL of 0.3 M CaCl_2_, was added and the pH was adjusted to 3 with 1 M HCl; distilled water was added until a final volume of 8 mL was reached, and the mixture was incubated at 37 °C for 2 h under agitation (130 rpm). Finally, 7.7 mL of simulated intestinal fluid (see Table 10) containing pancreatin (100 U/mL, based on trypsin activity) and bile salts (10 mM), 40 μL of CaCl_2_ 0.3 M, 0.15 mL of NaOH 1 M (to adjust pH to 7), and distilled water were added until a final volume of 14 mL was obtained and the mixture was incubated at 37 °C for 2 h under agitation (45 rpm).

At the end of the digestion process, the digested samples were centrifuged at 10,000 rpm for 10 min at 4 °C, and the supernatants were collected and stored at −20 °C until further analysis. Control samples without tortilla extracts were included. All treatments were performed in triplicate, resulting in 30 digestion assays. The bioaccessibility index (BI) was calculated according to the method described by Sánchez-Rodríguez et al. [70] as follows:

(2) $$BI\;\left(\%\right)=\frac{{TPC}_{I}}{{TPC}_{U}}\times 100$$

where TPC_I_ is the TPC after the intestinal phase and TPC_U_ is the TPC of the corresponding undigested extract.

### 3.15. Statistical Analysis

Three independent experimental replicates were prepared for each formulation and processing treatment. For tortillas, each independently prepared tortilla constituted one experimental unit. For baked and fried tortilla chips, one independently prepared masa sheet (8 tortilla chips) constituted one experimental unit; the individual chips obtained from the same masa sheet were considered subsamples for the corresponding analyses. All experimental analyses were conducted in triplicate (except tortilla and tortilla chip texture analyses) using a completely randomized design. Data from all analyses were evaluated by one-way analysis of variance (ANOVA) followed by Tukey’s post hoc test (*p* ≤ 0.05). Two complementary sets of one-way ANOVAs were performed. The first compared formulations (Control, F1, and F2) within each processing treatment (tortillas, baked tortilla chips, and fried tortilla chips). The second compared processing treatments within each formulation (Control, F1, and F2). Results were examined using SAS 9.0 statistical software.

## 4. Conclusions

The fortification of blue maize tortillas and tortilla chips with AF and QF improved their nutritional and functional properties while maintaining adequate technological quality. In general, the 9% fortification level (F2) exhibited the most favorable results, significantly increasing protein, mineral, phenolic, and antioxidant contents. Thermal processing strongly influenced phytochemical content and antioxidant activity, although these effects depended on the specific response evaluated. In general, baking maintained higher flavonoid content and antioxidant activity, while total phenolic content and bioaccessibility varied according to both processing method and formulation. Textural properties also varied according to formulation and processing conditions, with fortification improving tortilla flexibility while increasing hardness in tortilla chips, particularly in BTCs. During simulated gastrointestinal digestion, fortified products exhibited greater phenolic release than the controls, especially during the gastric phase. BTC-F2 maintained the highest phenolic content throughout digestion, whereas tortilla formulations exhibited the highest relative phenolic bioaccessibility. Overall, the findings demonstrate the potential of underutilized native species, such as ayocote and quintonil, to enhance the nutritional value of maize-based products while maintaining adequate technological quality. Furthermore, the present study establishes a foundation for future investigations that could include storage stability, sensory evaluation, identification and quantification of individual phenolic compounds, assessment of the antioxidant activity of the bioaccessible fraction, and in vivo validation of phenolic bioavailability, which would provide a more comprehensive understanding of their respective contributions to product quality and functionality.

## Figures and Tables

**Table 1:** Chemical composition of blue maize tortillas and tortilla chips (%).

Treatment	Formulation	Moisture *	Protein	Lipids	Ash	Carbohydrates	Crude Fiber	Caloric Intake *
Tortilla	C	50.38 ± 2.12 ^aA^	7.98 ± 0.19 ^cAB^	1.76 ± 0.12 ^aC^	1.89 ± 0.07 ^cA^	88.35 ± 0.11 ^aA^	2.50 ± 0.12 ^aC^	206.39 ± 7.83 ^aC^
	F1	51.39 ± 1.01 ^aA^	8.83 ± 0.10 ^bB^	1.61 ± 0.03 ^aC^	2.67 ± 0.03 ^bA^	86.89 ± 0.10 ^bA^	2.11 ± 0.05 ^bC^	199.99 ± 3.99 ^aC^
	F2	50.86 ± 1.35 ^aA^	10.43 ± 0.08 ^aA^	1.39 ± 0.03 ^bC^	2.82 ± 0.07 ^aA^	85.36 ± 0.02 ^cA^	2.21 ± 0.01 ^bB^	200.16 ± 5.41 ^aC^
BTC	C	6.75 ± 0.19 ^aB^	8.42 ± 0.03 ^bA^	3.61 ± 0.06 ^aB^	1.87 ± 0.02 ^bA^	86.10 ± 0.11 ^aA^	2.85 ± 0.04 ^bB^	382.84 ± 1.06 ^aB^
	F1	6.63 ± 0.18 ^aC^	10.12 ± 0.03 ^aA^	2.70 ± 0.05 ^bB^	2.69 ± 0.02 ^aA^	84.51 ± 0.10 ^bB^	2.90 ± 0.08 ^bB^	376.11 ± 0.36 ^bB^
	F2	6.59 ± 0.31 ^aC^	10.14 ± 0.03 ^aA^	2.80 ± 0.06 ^bB^	2.73 ± 0.02 ^aA^	84.36 ± 0.02 ^bA^	3.18 ± 0.04 ^aA^	376.59 ± 1.02 ^bB^
FTC	C	6.18 ± 0.15 ^cB^	7.57 ± 0.03 ^bB^	16.24 ± 0.64 ^aA^	1.47 ± 0.02 ^cB^	74.37 ± 0.12 ^abC^	3.84 ± 0.02 ^aA^	444.62 ± 5.95 ^aA^
	F1	8.29 ± 0.01 ^bB^	8.60 ± 0.03 ^aB^	13.90 ± 0.25 ^bA^	2.31 ± 0.01 ^bB^	75.02 ± 0.30 ^aC^	3.90 ± 0.19 ^aA^	421.45 ± 1.22 ^bA^
	F2	9.55 ± 0.09 ^aB^	8.53 ± 0.03 ^aB^	15.12 ± 0.28 ^aA^	2.44 ± 0.02 ^aB^	73.91 ± 0.49 ^bB^	3.02 ± 0.13 ^bA^	421.40 ± 1.11 ^bA^

* Wet basis; caloric intake calculated in kcal/100 g. Different lowercase letters in the same column indicate significant differences (*p* ≤ 0.05) between formulations of the same treatment. Different capital letters in the same column indicate significant differences (*p* ≤ 0.05) between the same formulation of different treatments. Values are expressed as the mean ± standard deviation (n = 3). C = control sample; BTC = baked tortilla chip; FTC = fried tortilla chip.

**Table 2:** Mineral composition of blue maize tortilla and tortilla chips (mg/100 g dry matter).

Treatment	Formulation	Ca	Fe	K	Mg	Mn	Na	Zn
Tortilla	C	215.70 ± 2.57 ^bC^	2.24 ± 0.21 ^aC^	475.95 ± 21.27 ^cC^	65.63 ± 4.99 ^bC^	0.31 ± 0.03 ^cB^	136.27 ± 13.86 ^aC^	6.47 ± 0.16 ^aC^
	F1	140.98 ± 5.77 ^cC^	1.10 ± 0.03 ^bB^	622.42 ± 20.62 ^bC^	62.82 ± 10.29 ^bC^	0.52 ± 0.03 ^aC^	95.88 ± 4.61 ^bC^	5.97 ± 0.23 ^aC^
	F2	244.09 ± 9.55 ^aC^	1.34 ± 0.08 ^bC^	776.86 ± 41.26 ^aC^	100.32 ± 3.95 ^aC^	0.43 ± 0.05 ^bB^	47.57 ± 3.71 ^bC^	5.01 ± 0.42 ^bC^
BTC	C	412.49 ± 11.99 ^bA^	5.17 ± 0.23 ^aA^	883.91 ± 35.70 ^cA^	122.73 ± 6.25 ^bA^	1.30 ± 0.11 ^aA^	248.99 ± 4.37 ^aA^	12.16 ± 0.50 ^aA^
	F1	265.53 ± 2.42 ^cA^	2.39 ± 0.12 ^cA^	1176.03 ± 36.11 ^bA^	117.44 ± 3.02 ^bA^	1.28 ± 0.05 ^aA^	180.26 ± 5.26 ^bA^	12.03 ± 0.39 ^aA^
	F2	459.20 ± 21.50 ^aB^	4.26 ± 0.15 ^bA^	1469.22 ± 57.22 ^aA^	194.58 ± 1.11 ^aA^	1.35 ± 0.05 ^aA^	85.94 ± 1.18 ^cA^	9.94 ± 0.21 ^bA^
FTC	C	340.15 ± 8.27 ^bB^	4.19 ± 0.20 ^bB^	749.57 ± 18.38 ^cB^	105.73 ± 8.17 ^bB^	0.38 ± 0.11 ^bB^	206.99 ± 7.24 ^aB^	10.16 ± 0.50 ^aB^
	F1	234.20 ± 2.61 ^cB^	2.29 ± 0.02 ^aA^	968.03 ± 21.54 ^bB^	97.44 ± 2.39 ^bB^	1.10 ± 0.09 ^aB^	154.93 ± 4.61 ^bB^	10.03 ± 0.39 ^ab^
	F2	378.54 ± 11.35 ^aA^	2.77 ± 0.35 ^aB^	1155.22 ± 29.48 ^aB^	153.24 ± 2.72 ^aB^	1.20 ± 0.09 ^aA^	72.27 ± 3.71 ^cB^	7.94 ± 0.21 ^bB^

Different lowercase letters in the same column indicate significant differences (*p* ≤ 0.05) between formulations of the same treatment. Different capital letters in the same column indicate significant differences (*p* ≤ 0.05) between the same formulation of different treatments. Values are expressed as the mean ± standard deviation (n = 3). C = control sample; BTC = baked tortilla chip; FTC = fried tortilla chip.

**Table 3:** Color parameters of blue maize tortilla and tortilla chips.

Treatment	Formulation	L*	a*	b*	C	h°
Tortilla	C	44.45 ± 0.17 ^aA^	1.22 ± 0.03 ^aB^	4.12 ± 0.19 ^cB^	4.28 ± 0.18 ^cB^	73.36 ± 0.56 ^cA^
	F1	42.56 ± 1.71 ^aA^	0.55 ± 0.20 ^bC^	8.80 ± 0.25 ^aB^	8.82 ± 0.03 ^aB^	86.02 ± 1.82 ^aA^
	F2	40.33 ± 0.46 ^bA^	0.70 ± 0.07 ^bC^	7.99 ± 0.13 ^bB^	8.01 ± 0.07 ^bB^	85.04 ± 0.59 ^bA^
BTC	C	37.08 ± 0.49 ^aB^	3.45 ± 0.17 ^aA^	7.17 ± 0.47 ^bB^	8.12 ± 0.56 ^bB^	60.50 ± 1.15 ^bB^
	F1	36.04 ± 0.40 ^aB^	4.56 ± 0.05 ^aB^	10.33 ± 0.38 ^aC^	11.24 ± 0.41 ^aC^	66.80 ± 0.52 ^aB^
	F2	32.36 ± 2.42 ^bB^	4.73 ± 1.05 ^aB^	10.29 ± 1.09 ^aC^	11.30 ± 1.44 ^aC^	65.90 ± 2.87 ^aB^
FTC	C	28.16 ± 1.00 ^aC^	3.66 ± 0.37 ^aA^	3.65 ± 0.29 ^bA^	5.16 ± 0.45 ^bA^	44.90 ± 1.44 ^bC^
	F1	28.42 ± 0.96 ^aC^	2.99 ± 0.11 ^bA^	5.41 ± 0.11 ^aA^	6.35 ± 0.28 ^aA^	60.53 ± 0.90 ^aC^
	F2	28.44 ± 0.42 ^aC^	2.76 ± 0.14 ^bA^	4.21 ± 0.60 ^bA^	5.16 ± 0.37 ^bA^	57.80 ± 1.23 ^aC^

Different lowercase letters in the same column indicate significant differences (*p* ≤ 0.05) between formulations of the same treatment. Different capital letters in the same column indicate significant differences (*p* ≤ 0.05) between the same formulation of different treatments. Values are expressed as the mean ± standard deviation (n = 3). C = control sample; BTC = baked tortilla chip; FTC = fried tortilla chip.

**Table 4:** Textural properties of masa.

Formulation	Hardness (N)	Adhesiveness (N s)	Cohesiveness	Elasticity (mm)
C	6.14 ± 0.11 ^a^	−0.23 ± 0.07 ^a^	6.19 ± 0.19 ^a^	0.309 ± 0.03 ^a^
F1	4.57 ± 0.19 ^b^	−1.16 ± 0.15 ^c^	5.10 ± 0.25 ^a^	0.325 ± 0.04 ^a^
F2	4.79 ± 0.09 ^b^	−0.8 ± 0.4 ^b^	5.55 ± 0.13 ^a^	0.335 ± 0.02 ^a^

Different lowercase letters in the same column indicate significant differences (*p* ≤ 0.05) between formulations of the same treatment. Values are expressed as the mean ± standard deviation (n = 3).

**Table 5:** Textural properties of blue maize tortillas.

Formulation	Young’s Modulus (MPa)	Tensile Strength (N)	Extensibility (%)	Rollability
C	0.161 ± 0.006 ^b^	1.70 ± 0.02 ^b^	12.29 ± 0.19 ^c^	1
F1	0.191 ± 0.013 ^a^	1.72 ± 0.02 ^b^	15.97 ± 0.25 ^a^	1
F2	0.185 ± 0.011 ^ab^	2.22 ± 0.04 ^a^	14.16 ± 0.13 ^b^	1

Different lowercase letters in the same column indicate significant differences (*p* ≤ 0.05) between formulations of the same treatment. Values are expressed as the mean ± standard deviation (n = 10).

**Table 6:** Textural properties of tortilla chips.

Treatment	Formulation	Hardness (gf)	Fracturability (mm)
BTC	C	1926.05 ± 122.12 ^bA^	2.97 ± 0.02 ^aA^
	F1	2436.38 ± 73.14 ^aA^	1.96 ± 0.05 ^cA^
	F2	1940.43 ± 142.73 ^bA^	2.26 ± 0.10 ^bA^
FTC	C	1723.95 ± 109.30 ^bB^	1.50 ± 0.14 ^aB^
	F1	2118.74 ± 111.85 ^aB^	1.56 ± 0.05 ^aB^
	F2	2010.73 ± 143.71 ^aA^	1.63 ± 0.04 ^aB^

Different lowercase letters in the same column indicate significant differences (*p* ≤ 0.05) between formulations of the same treatment. Different capital letters in the same column indicate significant differences (*p* ≤ 0.05) between the same formulation of different treatments. Values are expressed as the mean ± standard deviation (n = 9). C = control sample; BTC = baked tortilla chip; FTC = fried tortilla chip.

**Table 7:** Total phenolic content (TPC), total flavonoid content (TFC), and antioxidant activity of masa, blue maize tortillas and tortilla chips.

Treatment	Formulation	TPC (mg GAE/100 g ds)	TFC (mg QE/100 g ds)	DPPH (mg TE/100 g ds)	ABTS (mg TE/100 g ds)
Masa	C	82 ± 0.18 ^cA^	186 ± 0.07 ^bA^	186 ± 0.07 ^bA^	261 ± 0.24 ^cA^
	F1	94 ± 0.15 ^bA^	267 ± 0.14 ^aA^	214 ± 0.12 ^aA^	288 ± 0.32 ^abA^
	F2	105 ± 0.11 ^aA^	263 ± 0.12 ^aA^	210 ± 0.05 ^aA^	311 ± 0.27 ^aA^
Tortilla	C	76 ± 0.04 ^aAB^	100 ± 0.08 ^aC^	106 ± 0.12 ^aC^	2.17 ± 0.15 ^aB^
	F1	52 ± 0.04 ^bB^	39 ± 0.03 ^bC^	100 ± 0.05 ^bC^	210 ± 0.02 ^aB^
	F2	73 ± 0.07 ^aC^	93 ± 0.12 ^aC^	122 ± 0.10 ^aB^	200 ± 0.14 ^aB^
BTC	C	65 ± 0.01 ^bC^	192 ± 0.10 ^bA^	177 ± 0.03 ^bA^	232 ± 0.18 ^bAB^
	F1	95 ± 0.03 ^aA^	262 ± 0.23 ^aA^	212 ± 0.07 ^aA^	305 ± 0.15 ^aA^
	F2	100 ± 0.12 ^aA^	264 ± 0.25 ^aA^	211 ± 0.28 ^aA^	325 ± 0.11 ^aA^
FTC	C	69 ± 0.06 ^cBC^	138 ± 0.12 ^aB^	162 ± 0.08 ^aB^	162 ± 0.02 ^aC^
	F1	96 ± 0.09 ^bA^	133 ± 0.12 ^aB^	155 ± 0.14 ^aB^	151 ± 0.04 ^bC^
	F2	102 ± 0.03 ^aA^	132 ± 0.11 ^aB^	159 ± 0.09 ^aB^	151 ± 0.03 ^bC^

Different lowercase letters in the same column indicate significant differences (*p* ≤ 0.05) between formulations of the same treatment. Different capital letters in the same column indicate significant differences (*p* ≤ 0.05) between the same formulation of different treatments. Values are expressed as the mean ± standard deviation (n = 3). C = control sample; BTC = baked tortilla chip; FTC = fried tortilla chip.

**Table 8:** Changes in TPC (mg GAE/100 g ds) during in vitro bioaccessibility of blue maize tortillas and tortilla chips.

Treatment	Formulation	Digestion Phase			BI (%)
		Oral	Gastric	Intestinal	
Tortilla	C	113.26 ± 6.38 ^bA^	157.89 ± 13.6 ^bA^	41.10 ± 0.72 ^bA^	54.20 ± 3.53 ^cAB^
	F1	131.78 ± 4.54 ^aA^	227.65 ± 10.16 ^aA^	48.41 ± 1.67 ^aA^	93.54 ± 6.13 ^aA^
	F2	139.89 ± 4.75 ^aB^	244.42 ± 3.03 ^aB^	48.91 ± 3.15 ^aB^	71.63 ± 0.26 ^bA^
BTC	C	96.3 ± 2.6 ^cB^	150.49 ± 4.21 ^cAB^	38.39 ± 1.05 ^cAB^	58.74 ± 2.35 ^aB^
	F1	140.89 ± 2.52 ^bA^	225.29 ± 8.39 ^bA^	51.78 ± 1.82 ^bA^	55.17 ± 1.00 ^aB^
	F2	167.23 ± 9.59 ^aA^	273 ± 0.04 ^aA^	62.82 ± 3.51 ^aA^	65.03 ± 2.23 ^aA^
FTC	C	92.89 ± 4.25 ^bB^	133.12 ± 4.20 ^cB^	33.75 ± 3.08 ^bB^	50.39 ± 3.49 ^aB^
	F1	95.66 ± 3.78 ^bB^	150.71 ± 3.62 ^bB^	35.48 ± 1.73 ^bB^	38.24 ± 2.04 ^bC^
	F2	160.68 ± 6.71 ^aA^	193.78 ± 10.38 ^aC^	60.75 ± 2.75 ^aA^	59.36 ± 2.53 ^aB^

Different lowercase letters in the same column indicate significant differences (*p* ≤ 0.05) between formulations of the same treatment. Different capital letters in the same column indicate significant differences (*p* ≤ 0.05) between the same formulation of different treatments. Values are expressed as the mean ± standard deviation (n = 3). C = control sample; BTC = baked tortilla chips; FTC = fried tortilla chips.

**Table 9:** Formulation of the different fortified flours for tortilla/tortilla chips production.

Treatment	Formulation *		Ayocote:Quintonil Ratio	Nixtamalized Maize Flour (g)	Ayocote Flour (g)	Quintonil Flour (g)
Tortilla	C	0	-	16	-	-
	F1	6	1:1	15	0.5	0.5
	F2	9	2:1	14.6	0.94	0.46
BTC	C	0	-	16	-	-
	F1	6	1:1	15	0.5	0.5
	F2	9	2:1	14.6	0.94	0.46
FTC	C	0	-	16	-	-
	F1	6	1:1	15	0.5	0.5
	F2	9	2:1	14.6	0.94	0.46

* Nominal fortification level of the flour blend. F1 and F2 correspond to target fortification levels of 6% and 9%, respectively, with actual flour inclusions of 6.25% (*w*/*w*) and 8.75% (*w*/*w*) based on the total formulation; C = control sample; BTC = baked tortilla chip; FTC = fried tortilla chip.

**Table 10:** Preparation of simulated gastric fluids for in vitro digestion.

Salts	Simulated Oral Phase	Simulated Gastric Phase	Simulated Intestinal Phase
KCl 0.5 M (mL)	15.1	6.9	6.8
KH_2_PO_4_ 0.5 M (mL) NaHCO_3_ 1 M (mL)	3.7 6.8	0.8 12.5	0.9 42.5
NaCl 2 M (mL)	-	11.8	9.6
MgCl_2_·(H_2_O)_6_ 0.15 M (mL)	0.5	0.4	1.1
(NH_4_)_2_CO_2_ 0.5 M (mL)	0.06	0.5	-

## Data Availability

The data supporting the findings of this study are available within the article.
